# Sample size for detecting differentially expressed genes in microarray experiments

**DOI:** 10.1186/1471-2164-5-87

**Published:** 2004-11-08

**Authors:** Caimiao Wei, Jiangning Li, Roger E Bumgarner

**Affiliations:** 1 Department of Microbiology, University of Washington, Seattle, WA 98195, USA; 2 Department of Pathology, University of Washington, Seattle, WA 98195, USA

## Abstract

**Background:**

Microarray experiments are often performed with a small number of biological replicates, resulting in low statistical power for detecting differentially expressed genes and concomitant high false positive rates. While increasing sample size can increase statistical power and decrease error rates, with too many samples, valuable resources are not used efficiently. The issue of how many replicates are required in a typical experimental system needs to be addressed. Of particular interest is the difference in required sample sizes for similar experiments in inbred vs. outbred populations (e.g. mouse and rat vs. human).

**Results:**

We hypothesize that if all other factors (assay protocol, microarray platform, data pre-processing) were equal, fewer individuals would be needed for the same statistical power using inbred animals as opposed to unrelated human subjects, as genetic effects on gene expression will be removed in the inbred populations. We apply the same normalization algorithm and estimate the variance of gene expression for a variety of cDNA data sets (humans, inbred mice and rats) comparing two conditions. Using one sample, paired sample or two independent sample t-tests, we calculate the sample sizes required to detect a 1.5-, 2-, and 4-fold changes in expression level as a function of false positive rate, power and percentage of genes that have a standard deviation below a given percentile.

**Conclusions:**

Factors that affect power and sample size calculations include variability of the population, the desired detectable differences, the power to detect the differences, and an acceptable error rate. In addition, experimental design, technical variability and data pre-processing play a role in the power of the statistical tests in microarrays. We show that the number of samples required for detecting a 2-fold change with 90% probability and a p-value of 0.01 in humans is much larger than the number of samples commonly used in present day studies, and that far fewer individuals are needed for the same statistical power when using inbred animals rather than unrelated human subjects.

## Background

Microarray technology has become an important tool for studying gene expression levels on the whole genome scale [1]. One important objective of many microarray studies is to identify differentially expressed genes between different conditions. Despite the effectiveness of the technology, microarray experiments are usually done with very few replicates due to budgetary constrains, which often results in high false positive (Type I error) and false negative rates (Type II error). For many microarray experiments, once a list of genes has been identified, intensive follow-up investigations of these genes using traditional molecular tools are often pursued. Hence, valuable resources can be wasted in pursuing genes from experiments with a high false positive rate. Increasing the sample size increases the statistical power to detect expression differences in microarray analysis while also decreasing the error rate. However, it is important to balance sample size with other experimental goals so as not to waste resources. An important issue that concerns many biologists is, therefore "how many replicates are needed to obtain a given type of result?"

In general, the required sample size depends on the magnitude of the variability of the population, the magnitude of the expression change that is biologically meaningful (or desirable to detect), the power to detect the expression change, and the P-value/significance level/false positive rate. However, power and sample size have been viewed as complicated and difficult issues for microarray studies due to the large number of genes being investigated and little knowledge of the degree of natural expression variation within a population. To date, very few studies have assessed power and sample size requirements in microarray experiments. Pan et al. [2] proposed a normal mixture model to calculate the number of replicates required. In this study, the parameters were estimated using a subset of a real data set generated by cDNA arrays. This paper assumed that the replicates were independent of each other, whether they were drawn from the same individual or multiple individuals. Lee and Whitmore [3] discussed conceptual issues and presented computational methods (Analysis of Variance) for statistical power and sample size for different types of experimental designs, taking multiple testing into account. However, the data sets used to demonstrate these models contained a single pooled sample for each treatment/time point but not true biological replicates. Zien et al. [4] proposed a complex model that applies only to Affymetrix data to estimate biological variation and measurement error (normal additive and multiplication measurement error) for two sample comparisons. This study was based on 5 real Affymetrix data sets where the minimum required sample size was estimated based on a simulation study. Zien et al. [4] assumed a normal distributed additive measurement error and lognormal distributed measurement error. However, in real data, the functional form of the distribution of gene expression levels is generally unknown. The most common practice in microarray experiments is to assume normality of log transformed intensities or ratios. Pavlidis et al. [5] used a random sampling approach to evaluate the stability of the genes found to be differentially expressed between two groups from 16 published data sets; they found that the stability of some of the smaller data sets with fewer than 10 replicates was inconclusive. This approach is sound for the purpose of planning a study when pilot data is available with a large number of replicates. However, a pilot study is usually done with a small number of replicates where this is not feasible.

Pair-wise comparisons between conditions/groups/treatments are frequently used in microarray studies. Parametric and nonparametric statistical methods have been proposed to identify differentially expressed genes, among which t-tests are most commonly used. This paper is intended to provide some guidelines for sample size planning for pair-wise comparisons. Normalization is an essential and important pre-processing step in microarray data analysis. To our knowledge, no previous studies using multiple data sets have pre-processed the data sets in a comparable way. In addition, previous studies did not look at the effect of inbred vs outbred populations on the variation of gene expression. In order to make the results more comparable, we make use of 7 cDNA microarray data sets and apply the same normalization method (spatial lowess).

We estimate the variance by one sample t-test, paired t-tests or two sample t-tests on a gene-by-gene basis using several large expression data sets from both human, rats and mice. We then calculate the sample size required to detect a 1.5-, 2-, and 4-fold change in expression levels for the 90^th^, 75^th^, 50^th ^and 25^th ^percentile of genes ranked by variability at fixed settings for false positive and false negative rates. The sample size calculation provides the approximate but not exact number of replicates required for a given set of criteria.

## Results

### Data sets

We estimate the standard deviation and required sample size from 1 unpublished and 6 published cDNA data sets (Table 1). Data set A-C and E are from human samples. Data sets D and F-G are from mouse or rat samples. Data set F is from a study that is not yet published. The raw and/or pre-processed data for the unpublished dataset with gene order randomized and without the original gene/probe identifications can be downloaded from . Removing the gene IDs does not affect the analysis in this paper in any way. The same website also provides the links for the published data sets. In addition, all the control genes were removed from the data analysis for the cDNA data sets.

**Table 1:** cDNA microarray data sets used in the study

Data set	Reference	# Rep	# Genes	Tissue type	Description	Hybridization
A	Smith et al. 2003	20	15,592	Human liver	Paired HCC tumor vs adjacent non-tumor	Direct hyb between tumor and non tumor
B	Lapointe et al. 2004	41	38627	Human prostate	Paired prostate tumor vs adjacent non-tumor	Indirect hyb using common reference
C	Chen et al 2002	48	22618	Human liver	Paired HCC+HBV vs HBV	Indirect hyb using common reference
D	Pritchard et al. 2001	6	5281	Mouse liver and kidney	Paired liver vs kidney	Indirect hyb using common reference
E	Zhao et al. 2004	36 ductal + 21 lobular	44549	Human breast	lobular and ductal tumor tissue	Indirect hyb using common reference
F	NA	6	13, 056	Mouse liver	One third vs two thirds hepatectomy	Indirect hyb using individual baseline
G	Callow et al. 2000	8	5548	Mouse liver	ApoAI knock-out vs normal	Indirect hyb using common reference (pool)

Data set A comprises data generated from 40 liver RNA samples isolated from paired liver hepatocellular carcinoma (HCC) tumor and adjacent cirrhotic non-tumor tissue from 20 HCV infected Caucasian patients [6]. The objective of this study was to identify potential hepatocellular carcinoma markers. The microarray analysis was performed using in-house spotted human cDNA arrays containing 15,592 genes split between and spotted in duplicate on arrays HHD1 and HHD2. The duplicate sets of cDNAs within each slide were spotted side by side (panels A, B). RNA from tumor and adjacent non-tumor tissue from individual patients was co-hybridized to 2 slides (one was a dye flip of the other).

Data set B was generated from 41 matched pairs of prostate tumor and non-tumor tissue hybridized to arrays spotted with 38627 cDNAs. All samples were labeled with Cy5 and co-hybridized with a common reference labeled in Cy3 [7]. The purpose of this study was to identify the difference in expression levels between normal and prostate tumor tissues.

Data set C was generated from RNA isolated from paired HCC tumor and adjacent non tumor liver from 41 HBV infected patients [8,9] hybridized to cDNA arrays. Each RNA sample was labeled with either Cy5 and co-hybridized with Cy3-labeled reference RNA. The purpose of this study was to identify gene expression differences between HCC tumor and non-tumor liver tissue.

Data set D was generated from RNA isolated from paired liver and kidney tissue from 6 male C57BL6 mice [9] hybridized to cDNA arrays. Each RNA sample was labeled with Cy5 and co-hybridized with Cy3-labeled amplified RNA from Universal Human Reference to total RNA (Stratagene). The purpose of this study was to identify gene expression differences between tissue types.

Data set E was generated from RNA isolated from 36 breast ductal tumor and 16 lobular tumor tissues [10] hybridized to cDNA arrays. Each RNA sample was split in two and labeled with either Cy5 or Cy3, and co-hybridized with a common reference RNA as color flips with two replicates (4 arrays/tissue/mouse). The purpose of this study was to identify gene expression differences between ductal carcinoma and lobular carcinoma.

Data set F consists of data generated from 24 liver tissue samples from 12 inbred mice (unpublished data). One third or two thirds of the liver was removed from each mouse and used as the baseline samples. At 12 hours post operation, the mice were sacrificed and the remaining liver tissue was used as the experimental sample. The aim of this study was to screen for genes potentially related to liver regeneration after hepatectomy. RNA samples from the 12 hour post-operation livers were co-hybridized with their own baseline liver samples. A total of four DNA arrays were used for each sample comparison. Two sets of arrays (MOD1 and MOD2), each containing 6528 different cDNAs spotted in duplicate (A and B) on each array were used. In addition, each comparison was done with a dye flip pair of slides. This data set made use of arrays generated at the University of Washington Center for Expression Arrays.

The goal of data set G was to identify genes with altered expression in the liver tissues of two mouse models with very low HDL cholesterol levels (treatment groups) as compared to inbred control mice. The mouse model considered in this study is the Apolipoprotein AI (ApoAI) knock-out, where ApoAI is a gene known to play a pivotal roles in HDL metabolism [11,12]. Each cDNA array contained 5548 non-control genes or ESTs. A pool of normal RNA samples labeled with Cy3 served as the reference for all the arrays.

In summary, three of the data sets (D, F-G) are from inbred mouse and rat strains respectively, and the other four data sets (A-C, and E) are from large scale studies of gene expression in humans. If all other factors (assay protocol microarray platform, data pre-processing) were equal, one might anticipate that fewer individuals would be needed for the same statistical power using inbred animals as opposed to unrelated human subjects.

### Background adjustment and normalization

Background adjustment and normalization is necessary to remove systematic biases of non-biological origin in microarray studies. A number of methods of background correction and normalization have been proposed [13,14]. We used the locally written program "spot-on Image" to analyze the cDNA array data for data sets A and F. Spot-on uses a local background for each spot. The background subtracted intensity of all cDNA data sets were normalized by the spatial lowess method using the R add on package MAANOVA written by the Jackson Lab, which is available at .

### Estimates of standard deviation and sample size calculation

**Figure 1:**
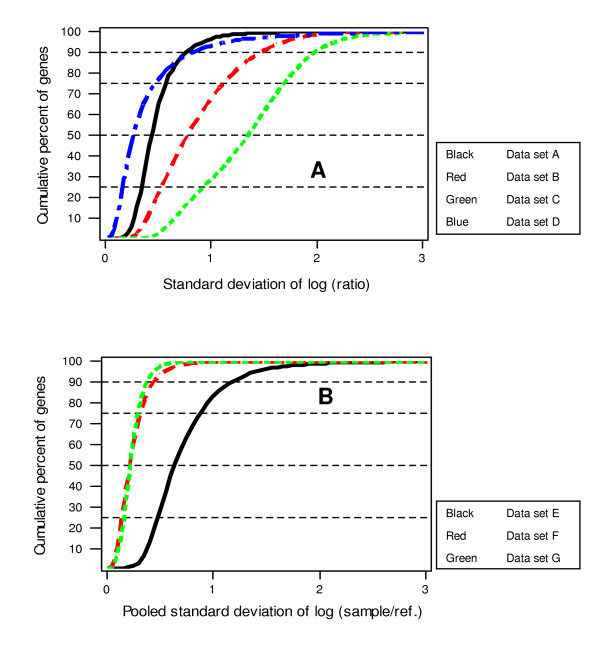
Histogram of standard deviation The X axis is the standard deviation, and the Y axis is the percentage of genes that has standard deviation below the value of X. All data sets were normalized by spatial lowess; (A) Data set A-standard deviation of log ratio of two groups (direct hybridization); data set B-D standard deviation of the difference of log (sample/reference) of the two groups (indirect hybridization); (B) Data sets E-G common standard deviation of (sample/reference) of the two independent groups (indirection hybridization).

The distribution of the standard deviations estimated from these 7 data sets are presented in Figure 1. All data are log_2 _transformed prior to data analysis. Figure 1A shows the standard deviation of the log ratio of the 4 paired cDNA data sets (A-D). The standard deviations of data sets E-G in Figure 1B are the common standard deviation of the log_2 _ratio (sample/reference) of two independent groups.

The required sample size of an experiment depends on the variance component (*σ*), the desired detectable fold change (*δ*), the power to detect this change (1-*β*, the likelihood of detecting the change or the true positive rate), and a chosen type I error rate (*α*). For microarrays, a combination of fold-change and test p-value is commonly used for selecting differentially expressed genes between two groups or conditions. In this study, sample sizes were calculated in R using the function of power.t.test. The required input parameters are the log scale fold change of interest *δ *(*δ *= 1 in log transformed data translates into a 2 fold change in expression level, *δ *= 2 in log transformed data translates into a 4 fold change in expression level, etc), significance level, power, and the standard deviation (common standard deviation for two sample t-test, the standard deviation of the difference within subject for paired t-test, or the standard deviation of one sample t-test), the type of t-tests (one sample, two sample, or paired t-test), and the type of test (two sided or one sided).

For example, in the case of data set A (one sample t-test), if we wish to find out the approximate sample size to detect a 2 fold change (*δ *= 1) in expression level between tumor and non-tumor tissue in the 75% least variable genes (*σ *<= 0.5884) with a two sided 0.001 significance level test with 90% power, we could use the following R function

power.t.test(n = NULL, delta = 1, sd = 0.5584, sig.level = 0.001, power = 0.9, type = "one.sample", alternative = "two.sided")

Where sd = 0.5584 is the 75^th ^percentile of the standard deviation of log ratio.

In the case of data set G (two sample t-test), if we wish to find the approximate sample size to detect a 2 fold change (*δ *= 1) in expression level between knock-out and control mice in the 75% least variable genes (*σ *<= 0.3102) with a two sided 0.001 significance level test with 90% power, we could use the following R function

power.t.test(n = NULL, delta = 1, sd = 0.3102, sig.level = 0.001, power = 0.9, type = "two.sample", alternative = "two.sided")

Where sd = 0.3102 is the 75^th ^percentile of the common standard deviation of log (sample/reference).

In R, for a one sample t-test or a paired t-test to have power 1-*β *to reject for a two sided testing and strict interpretation of tail probability with significance level *α *for detecting a difference of *δ*, the minimum number of samples or pairs is obtained by solving the following equation iteratively

Power = Pr(t_v, ncp _< t_v, *α*/2_) + Pr(t_v, ncp _> t_v, 1-*α*/2_)

Where ncp is the noncentrality parameter of the non-central t-distribution, and is estimated by



t_v, *α*/2 _is the *α*/2 quantile of a central t-distribution with v degrees of freedom and v = n-1. t_v, ncp _follows a non-central t-distribution with v degrees of freedom and a non-centrality parameter of ncp.

For a two sample t-test with equal sample sizes, if we wish to have a large enough sample to detect a difference *δ *(with a two-sided test and strict interpretation of tail probability with *α *significance level test with 1-*β *power), then the sample size (n) for each group is obtained by solving the following equation iteratively

Power = Pr(t_v, ncp _< t_v, *α*/2_) + Pr(t_v, ncp _> t_v, 1-*α*/2_)

Where ncp is the noncentrality parameter of non-central t-distribution, and is estimated by



t_v, *α*/2 _is the *α*/2 quantile of a central t-distribution with v degrees of freedom and v = 2n - 2. t_v, ncp _follows a non-central t-distribution with v degrees of freedom and a non-centrality parameter of ncp.

**Figure 2:**
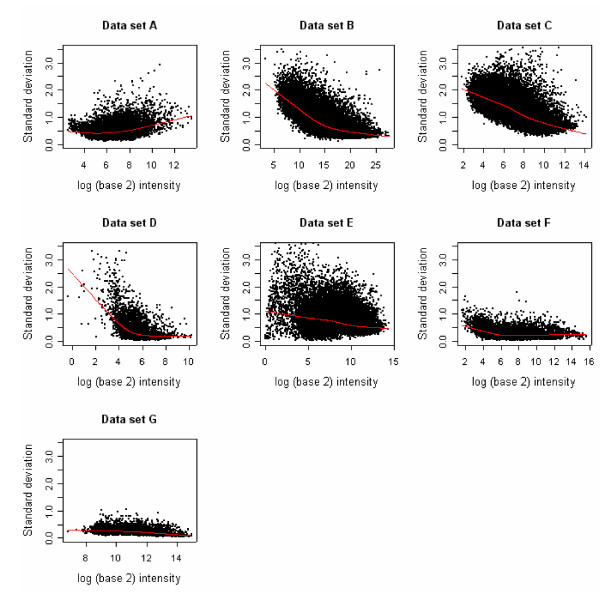
Standard deviation versus log intensity Standard deviations are based on one sample t-test (data set A), paired t-test (data sets B-D), or two independent t-test (data sets E-G).

Microarray experiments usually involve a large number of genes, with variance components varying greatly across the genes. In general, the variance is higher for low expressors which make up of a large percentage of the genes (Figure 2). Approximately 50% of genes are called absent on the Affymetrix full genome GeneChips. It is reasonable to choose a value of variance, e.g. the median or the upper 75^th ^percentile of variance across all genes, and to use this as the value in the power calculations. For example, if we use the variance for the 50^th ^percentile, then the sample size calculations will assure us of having the desired power to detect a chosen n-fold change for all but the 50% most variable genes. In Figure 1, we show horizontal lines at the 25^th^, 50^th^, 75^th ^and 90^th ^percentiles. The intersection of these lines with the "cumulative percentage of genes" provides the value of *α *for each data set. Additional file 1 shows the estimated sample size required to detect a 1.5-, 2-, and 4-fold change in expression level for the 90^th^, 75^th^, 50^th^, and 25^th ^percentile genes for a given setting of false positive rate and power. As is expected, the required sample size increases with increasing variance, increasing power, and decreasing fold-change and false positive rate.

A significance level (the probability of making a type I error, that is getting a false positive) of 0.05 is often employed in hypothesis testing. Thousands of genes are usually studied in microarray experiments. When more than 10,000 genes are tested independently, we would expect more than 500 genes to appear as false positives when the 0.05 significance level is applied. Hence, a smaller cut-off p-value should be used in order to reduce the number of false positives. Many multiple testing correction methods have been proposed. The simplest one is the Bonferroni correction (family wise error control) where the nominal significance level is divided by the number of tests. The Bonferroni correction is very stringent. False discovery rate (FDR) [15], the proportion of false positives among the genes that are identified as differentially expressed, is a post-data measure for controlling false positive. For the purpose of sample size planning, we suggest using the family wise type of error control. A higher false positive rate (lower false negative rate) can be employed for studies aiming to eliminating non-significant differentially expressed genes, and a smaller false positive rate can be used for those studies involving costly follow-up research.

For reference, Table 2 lists the number of genes/ESTs/probes found to be differentially expressed between two conditions by different significance levels and fold changes. The number of significant genes in data sets F and G are small, possibly due to the relatively fewer number of genes included in the study and the homogeneity between conditions (data set F compare in-bred mice with two different volumes of hepatectomy; data set G compares ApoAI knock-out versus normal mice).

**Table 2:** Significant genes/ESTs/probes called by methods used in the studies using different criteria (combination of significance level and fold changes)

Data set	Reference	P <= 0.001 and the estimated fold change >=2	P <= 0.001 only	P <= 0.01 only
A	Smith et al. 2003	183	1783	3590
B	Lapointe et al. 2004	609	6549	10153
C	Chen et al 2002	1253	4187	6197
D	Pritchard et al. 2001	479	1557	1845
E	Zhao et al. 2004	270	1050	3821
F	NA	16	145	723
G	Callow et al. 2000	6	11	77

## Discussion

Factors that affect sample size calculation include the magnitude of the variability of the population, the magnitude of the desired detectable expression change, the chosen power to detect the expression change, and the cut-off P-value/significance level/false positive rate. For a given study, the variability of the population being studied is fixed, and once researchers have identified the desired detectable expression change, the required sample size depends on the chosen false positive and false negative rates. The variability of human subject data is typically larger than that seen with laboratory animals and cell lines due to genetic influences on gene expression. Hence, more replicates are needed for studies that involve human subjects (or any other outbred population) than for studies with samples from an inbred population. This is readily apparent in the cDNA data in Additional file 1 (data sets A-C, and E are human samples while D and F-G are from mice). With the cDNA array data, one needs roughly 5 times as many human samples relative to mouse to detect the same magnitude of change with the same statistical power at the same significance level. This increase in the required number of samples for an outbred population has not been discussed before and has practical implications for those wishing to translate gene expression work from animal models to studies in human populations.

Multiple levels of replicates are common in two color microarray experiments. Multiple arrays probed with RNA samples isolated from multiple individuals of a population/treatment/group are referred to as biological replicates. Multiple arrays hybridized using the same RNA or multiple replicates of the same gene within an array are referred to as technical replicates. Although technical replicates can improve the precision and the reliability of the measurement and provide information for quality control, biological replicates are most effective in reducing the variance of the estimate of mean difference. Biological replicates therefore increase the power to detect biologically significant gene expression differences. More importantly, when trying to identify differences between a treatment and a control group, accurate estimates of the biological variability within the groups is essential to determine if the between group differences are meaningful (by a t-test, Analysis of Variance (ANOVA) or other method).

Careful experimental design is necessary to maximize the statistical power of the test [16-18] while balancing resource allocation. For example, dye swapping (in which pairs of RNA samples are hybridized twice with reverse dye labeling) is common in two color array experiments and is a great help in removing dye bias. However, if the experiments involve a common reference sample, which is not of biological interest and the goal is to identify gene expression differences between two groups (both of which are co-hybridized again the common reference), using twice as many independent biological replicates is preferable to dye swapped technical replicates.

### Caveats

This paper is intended to give some guidance to those planning microarray experiments. The sample size calculations we performed provide an approximate number of replicates for a given set of criteria. Our studies were limited to a small number of published microarray studies for which the following criteria were true:

1) A reasonably large number of biological replicates were analyzed.

2) Raw data was readily available so that we could reprocess all data with the same algorithms.

3) Other potentially large sources of variability such as flow sorting, laser micro-dissection and/or multiple rounds of amplification were not present.

We have only analyzed date from a limited number of tissue types – liver, prostate, breast and blood in human, liver and kidney in mouse, and mammary gland in rat. It is entirely possible that different tissue types will have larger or smaller degrees of biological variation and hence will require more or fewer samples to reach a given conclusion. In addition, lab or experiment specific methods of obtaining and processing samples may induce greater degrees of expression variation than seen in our sample data. As more large data sets become available, it will be useful to extend these studies to better define the magnitude of gene expression variation in purebred animals and in outbred humans across a variety of tissues.

However, the data shown in Additional file 1 however should be sobering to those planning or reviewing an experimental protocol for microarray analysis. In these limited data sets with human samples hybridized to cDNA arrays with a common reference (B-C, E), we show that 32 samples for each group are required to detect a 2-fold change in the 75% least variable genes with 90% power and a p-value of 0.001. With a p-value of 0.01 and 90% power, at least 20 samples are required to detect a 2-fold change in the 75% least variable genes. This is a much larger number of samples than is frequently used in human microarray case-control studies designed to identify gene expression differences between two groups.

## Methods

### Data set selection, pre-processing and normalization

Background adjustment and normalization is needed in microarray data analysis in order to remove non-biological variation. Intensity based normalization methods such as locally weighted least square polynomial regression (lowess) is commonly used in cDNA microarray experiments. The background subtracted intensities were normalized by the spatial lowess method using the R add on package MAANOVA written by the Jackson Lab. For the two cDNA experiments with replicate panels within each array, we normalized the two panels separately. All control genes were excluded from data analysis for data sets A-G.

### Estimate of variance components

Pair-wise comparisons among conditions/groups/treatments of gene expression levels are common goals of microarray studies. Simultaneous comparison of more than two treatments/conditions using one way ANOVA can be advantageous. However, a significant F for a comparison of several treatments does not provide information about which particular groups differ from each other. In addition, one way ANOVA is not sensitive to treatment effects when only one or two samples out of many are quite different. T-tests are commonly used to compare individual treatments in pairs.

In order to calculate power and plan sample size, one must first estimate the variance. We applied paired or two sample t-tests in this study based on the correlation between the two groups. For data set A, as the pairs of tumor and adjacent non-tumor tissue are highly correlated, we used two tailed one sample t-tests with the normalized log_2 _ratio of tumor/non-tumor as the response variable. Data sets B-D were generated from paired samples using a reference design on cDNA arrays; Paired t-tests are appropriate for these three data sets. We performed two tailed, two sample (or independent) t-tests on data sets E-G with normalized log ratio as the response variable. The two sample t-tests are based on unequal variances for the two groups of samples.

The variances of the data sets with paired samples are the variance of the difference. The common variance of the datasets with independent samples was estimated by the following formula:



Where n_1_, n_2 _are the number of observations for group 1, and group 2, respectively; and S_1 _and S_2 _are the standard deviation for group 1, and group 2, respectively.

To simplify power and sample size calculation, and to focus our calculation on *biological *variance, the log ratios of the 4 technical replicates of data sets A, E, and G were averaged for each RNA pair (data set A) or sample (data sets E and G). The standard deviations across biological replicates were estimated on a gene-by-gene basis. Sample sizes were calculated using R for detecting a 1.5-, 2- or 4-fold change for the 90%, 75%, 50%, and 25% least variable genes with a range of power (0.70, 0.80, 0.90) and confidence level (0.01, 0.001, 0.0001), assuming equal sample size for the two groups. The numbers for sample size are rounded to integers.

## Authors' contributions

JL provided the mouse liver microarray data set F prior to publication. CW performed all the analysis in this paper. RB supervised JL and CW and contributed to the design, coordination and writing. All authors read and approved of the final manuscript.

## Supplementary Material

Additional File 1 Sample size required to detect a 1.5-, 2-, and 4-fold changes of expression level for the 90%, 75%, 50%, and 25% least variable genes for a given settings of false positive rates (*α*) and power (1-*β*). This additional file shows the estimated sample size to a 1.5-, 2, and 4-fold changes of expression level for the 90%, 75%, 50%, and 25% least variable genes for a given settings of false positive rates (*α*) and power (1-*β*) for all of the data sets referred in Table 1. Click here for file
