# Supplementary material for: Sample size for detecting differentially expressed genes in microarray experiments
Source: BMC Genomics. 2004 Nov 8;5:87. doi: 10.1186/1471-2164-5-87 (PMC533874; doi:10.1186/1471-2164-5-87)
Supplement: Additional File 1 — Sample size required to detect a 1.5-, 2-, and 4-fold changes of expression level for the 90%, 75%, 50%, and 25% least variable genes for a given settings of false positive rates (α) and power (1-β). This additional file shows the estimated sample size to a 1.5-, 2, and 4-fold changes of expression level for the 90%, 75%, 50%, and 25% least variable genes for a given settings of false positive rates (α) and power (1-β) for all of the data sets referred in Table 1. [file 1471-2164-5-87-S1.pdf]

Sample size required to detect a 1.5-, 2-, and 4-fold changes of expression level for the 90%, 75%, 50%, and 25% least variable genes for a given settings of false positive rates (α) and power (1-β)

Standard Deviation(σ)	1-β	1.5-fold change			2-fold change			4-fold change		
		α=0.0001	α=0.001	α=0.01	α=0.0001	α=0.001	α=0.01	α=0.0001	α=0.001	α=0.01
**Data set A**										
0.7808	0.9	54	42	29	24	18	13	11	8	6
(90 percentile)	0.8	47	35	24	21	16	11	10	7	5
	0.7	42	31	20	19	14	9	9	7	5
0.5884	0.9	34	26	18	16	13	9	9	7	5
(75 percentile)	0.8	30	22	15	15	11	7	8	6	4
	0.7	27	20	13	14	10	7	8	6	4
0.4556	0.9	23	18	12	12	9	7	7	6	4
(50 percentile)	0.8	21	15	10	11	9	6	7	5	4
	0.7	19	14	9	11	8	5	6	5	3
0.3559	0.9	17	13	9	10	8	5	6	5	4
(25 percentile)	0.8	15	11	8	9	7	5	6	5	3
	0.7	14	10	7	9	6	4	6	4	3
**Data set B**										
1.4809	0.9	176	137	97	66	51	36	22	17	12
(90 percentile)	0.8	149	113	77	57	43	29	19	15	10
	0.7	130	97	64	50	37	24	18	13	9
1.1322	0.9	106	83	58	42	32	22	16	12	8
(75 percentile)	0.8	90	68	46	36	27	18	14	11	7
	0.7	79	59	39	32	24	16	13	10	6
0.7926	0.9	56	43	30	24	19	13	11	8	6
(50 percentile)	0.8	48	36	24	21	16	11	10	8	5
	0.7	43	32	21	19	14	9	9	7	5
0.5468	0.9	30	23	16	15	11	8	8	6	4
(25 percentile)	0.8	27	20	13	14	10	7	8	6	4
	0.7	24	18	12	13	9	6	7	5	4
**Data set C**										
1.9985	0.9	315	246	174	114	89	63	34	26	18
(90 percentile)	0.8	265	202	138	97	74	50	30	22	15
	0.7	231	173	114	85	64	42	27	20	13
1.7050	0.9	231	180	128	85	66	47	27	21	14
(75 percentile)	0.8	195	148	101	73	55	37	24	18	12
	0.7	171	127	84	64	48	31	21	16	10
1.3650	0.9	151	117	83	57	44	31	20	15	10
(50 percentile)	0.8	128	97	66	49	37	25	17	13	9
	0.7	112	83	55	44	32	21	16	12	8
0.9470	0.9	77	59	42	31	24	17	13	10	7
(25 percentile)	0.8	65	49	33	27	21	14	12	9	6
	0.7	58	43	28	25	18	12	11	8	5

Standard Deviation(σ)	1-β	1.5-fold change			2-fold change			4-fold change		
		α=0.0001	α=0.001	α=0.01	α=0.0001	α=0.001	α=0.01	α=0.0001	α=0.001	α=0.01
**Data set D**										
0.8383	0.9	62	48	33	26	20	14	11	9	6
(90 percentile)	0.8	53	40	27	23	17	12	10	8	5
	0.7	47	35	23	21	15	10	10	7	5
0.4786	0.9	25	19	13	13	10	7	7	6	4
(75 percentile)	0.8	22	17	11	12	9	6	7	5	4
	0.7	20	15	10	11	8	5	7	5	4
0.2801	0.9	13	10	7	8	6	4	6	4	3
(50 percentile)	0.8	12	9	6	8	6	4	5	4	3
	0.7	11	8	5	7	6	4	5	4	3
0.1801	0.9	9	7	5	6	5	4	5	4	3
(25 percentile)	0.8	8	6	4	6	5	3	5	4	3
	0.7	8	6	4	6	4	3	4	3	3
**Data set E**										
1.1985	0.9	225	175	125	81	63	44	23	18	12
(90 percentile)	0.8	189	144	98	68	52	35	20	15	10
	0.7	165	123	81	60	45	29	18	13	9
0.8985	0.9	128	100	71	47	36	26	15	11	8
(75 percentile)	0.8	108	82	56	40	30	21	13	10	7
	0.7	94	70	46	35	26	17	12	9	6
0.6615	0.9	71	55	39	27	21	15	10	7	5
(50 percentile)	0.8	60	46	31	23	18	12	9	7	4
	0.7	53	39	26	21	15	10	8	6	4
0.5001	0.9	42	33	23	17	13	9	7	5	4
(25 percentile)	0.8	36	27	19	15	11	8	6	5	3
	0.7	32	24	16	14	10	7	6	5	3
**Data set F**										
0.4607	0.9	36	28	20	15	12	8	7	5	4
(90 percentile)	0.8	31	24	16	13	10	7	6	5	3
	0.7	28	21	13	12	9	6	6	4	3
0.3328	0.9	21	16	11	10	7	5	5	4	3
(75 percentile)	0.8	18	14	9	9	7	5	5	4	3
	0.7	16	12	8	8	6	4	4	3	2
0.2362	0.9	12	10	7	7	5	4	4	3	2
(50 percentile)	0.8	11	8	6	6	5	3	4	3	2
	0.7	10	7	5	6	4	3	4	3	2
0.1672	0.9	8	6	4	5	4	3	3	3	2
(25 percentile)	0.8	7	6	4	5	4	3	3	3	2
	0.7	7	5	3	4	3	2	3	3	NA

Standard Deviation(σ)	1-β	1.5-fold change			2-fold change			4-fold change		
		α=0.0001	α=0.001	α=0.01	α=0.0001	α=0.001	α=0.01	α=0.0001	α=0.001	α=0.01
**Data set G**										
0.3971	0.9	28	22	15	12	9	7	6	4	3
(90 percentile)	0.8	24	18	12	11	8	6	5	4	3
	0.7	21	16	10	10	7	5	5	4	3
0.3102	0.9	19	14	10	9	7	5	5	4	3
(75 percentile)	0.8	16	12	8	8	6	4	4	3	3
	0.7	15	11	7	7	6	4	4	3	2
0.2412	0.9	13	10	7	7	5	4	4	3	2
(50 percentile)	0.8	11	9	6	6	5	3	4	3	2
	0.7	10	8	5	6	4	3	4	3	2
0.1867	0.9	9	7	5	5	4	3	4	3	2
(25 percentile)	0.8	8	6	4	5	4	3	3	3	2
	0.7	8	6	4	5	4	3	3	3	2

Sample size is the number of pairs of samples or the number of sample for each group for independent samples. NA indicates the sample size can not be estimated.
